# Correlation of X-Ray Vector Radiography to Bone Micro-Architecture

**DOI:** 10.1038/srep03695

**Published:** 2014-01-15

**Authors:** Florian Schaff, Andreas Malecki, Guillaume Potdevin, Elena Eggl, Peter B. Noël, Thomas Baum, Eduardo Grande Garcia, Jan S. Bauer, Franz Pfeiffer

**Affiliations:** 1 Department of Physics and Institute of Medical Engineering, Technische Universität München, Germany; 2 Institut für Radiologie, Klinikum rechts der Isar, Technische Universität München, Germany; 3 Abteilung für Neuroradiologie, Klinikum rechts der Isar, Technische Universität München, Germany

## Abstract

Besides the overall mass density, strength of trabecular bone depends significantly on its microstructure. However, due to dose constraints in medical CT imaging, it is impossible to gain sufficient information about very fine bone structures in vivo on the micrometer scale. Here we show that a recently developed method of X-ray vector radiography (XVR), an imaging method which uses X-ray scattering information to form an image, allows predictions on the bone microstructure without the explicit need to spatially resolve even individual trabeculae in the bone. We investigated thick human femoral bone samples and compared state-of-the-art *μ*CT data with XVR imaging. A model is presented which proves that XVR imaging yields information directly correlated with the trabecular microstructure. This opens up possibilities of using XVR as a tool to help early diagnosis of bone diseases, such as osteoporosis.

Osteoporosis is an under-diagnosed, serious bone disease that affects a substantial amount of the population. Estimated 200 million people worldwide are affected by osteoporosis today^1^. An early diagnosis is crucial to ensure a proper treatment before fractures might occur. The gold standard used in today's diagnostics of osteoporosis is dual-energy X-ray absorptiometry (DXA) and the T-score value calculated from the resulting bone mineral density (BMD)^2^. One often recommended location for diagnosis is the proximal femur area^3^,^4^,^5^,^6^, as it is subject to large mechanical loads and poses an area of high risk for a fracture when suffering from osteoporosis. Even though DXA is able to assess bone quality to some degree, it is only able to give information about the BMD, whereas bone strength also depends on other factors, such as contributions caused by its structure^7^.

Grating-based phase-contrast X-ray imaging is a recently developed method that is able to provide complementary information compared to conventional radiography^8^,^9^,^10^. Using grating-based X-ray imaging, one can also obtain information about the local scattering strength of microstructures within a sample on a scale smaller than the actual pixel size. In analogy to visible light microscopy, this has been called dark-field X-ray imaging^11^. The recorded small angle scattering (SAS) signal often varies under rotation of the sample and we have previously called this direction-dependent dark-field imaging X-ray vector radiography (XVR)^12^. As the microstructure of the trabeculae plays an essential role in determination of bone strength (e.g. in pathologies like osteoporosis), we used trabecular bone in this study^12^. In bone research the so-called degree of anisotropy (DA_B_) is an important parameter to characterize the structural properties of trabcular bone^13^,^14^,^15^,^16^. One property of the XVR signal is that it often shows a preferred scattering orientation and thus an anisotropy of the signal can be measured^17^. To avoid confusion, the term anisotropy will always refer to the anisotropy of the XVR signal (DA_XVR_) in this article.

We have previously shown that thin slices of human trabecular bone do indeed display an anisotropic scattering behaviour under rotation and that XVR is able to record the different scattering orientations. In contrast to absorption based imaging modalities, such as *μ*CT, XVR maintains its ability to give structural information even at coarse resolution and consequently low radiation exposure^12^. Naturally, human bone does not only consist of thin slices, but rather of a complex assembly of trabeculae in various directions. While it is possible to obtain information about this trabecular structure ex-vivo using *μ*CT, this method is not suitable for diagnostics due to the very high dose required to directly resolve the individual trabeculae in great detail. For possible use of XVR as a diagnostical tool, it is vital to have a good understanding of how the XVR signal is being formed within a whole volume of trabecular bone. In this study we compare state-of-the-art *μ*CT of thick human femoral bone samples with the respective XVR results.

Figure 1 sketches the fundamental differences between a *μ*CT system and an experimental XVR setup. For the remainder of this article, we use a Cartesian coordinate system with its z-axis parallel to the propagation direction, y-axis parallel to the grating lines and the x-axis orthogonal to both of them. By using an elaborate numerical analysis, we correlate the XVR signal to the present trabecular microstructure within the sample.

## Results

In analogy to conventional absorption-based imaging, which is governed by the Lambert-Beer-Law, the visibility signal recorded during an XVR scan, *V*, can be described with an exponential function. The oscillating term is used to include anisotropic scattering within the sample^18^: 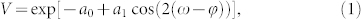


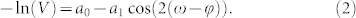
In eq. (1) and (2), (*ω* − *φ*) is the relative angle between the grating lines and the main scattering orientation in the sample. The degree of anisotropy (*DA*_XVR_) of the XVR signal can be defined as the amplitude of the oscillating part (*a*_1_) to the constant part (*a*_0_), which by this definition assumes values between 0 and 1: 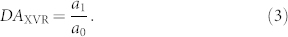
For our measurements, human femur head bone samples were acquired from three different donors at the pathological institute of Klinikum Rechts der Isar, Munich. Eight cubes (P1–P8) of 1 cm^3^ each were cut out around a defined axis through the centre of the femoral head as shown in fig. 2(a). Furthermore, a 3-D *μ*CT scan rendering (b) as well as two cuts (c,d) are displayed. In agreement with local laws and institutional requirements, the samples were harvested to be used for educational and research purposes in consent with their respective donors.

In the following, our aim is to connect the X-ray vector radiography signal to the actual microstructure. Starting from raw high-resolution CT absorption data from trabecular bone samples, we have a three dimensional volume of linear attenuation coefficients *μ*(*x*, *y*, *z*). We assume that scattering occurs mainly at borders between different materials in the sample, i.e. bone and soft tissue/air, which are perpendicular to the incident X-ray beam. In the case of the investigated bones, the different materials could be easily distinguished by their linear absorption coefficient since the predominant materials were bone, soft tissue and air.

From the two gradients of *μ_n_* perpendicular to the incident beam, *G_x_*_,*n*_ and *G_y_*_,*n*_, we can calculate the direction of biggest change *φ_n_*(*x*, *y*) and its magnitude *A_n_*(*x*, *y*) in different planes *n*: 
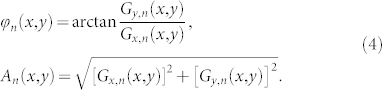
Using the superposition principle for XVR imaging^18^,^19^ to add the individual planes, we can calculate a function *ψ*(*x*, *y*), from which the orientation and anisotropy of the XVR signal can be derived: 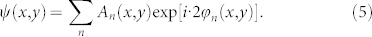
An elaborate description of the model is given in the Methods section.

XVR measurements were performed on all eight cubes from three orthogonal sides each, resulting in a grand total of 24 independent scans. Likewise, the described model was applied to the *μ*CT data along the three corresponding planes for each sample. A comparison between these two methods for specimen P4 can be seen in fig. 3. The main structural orientation is coded in the colour wheel, i.e. a horizontal structure that scatters in the vertical direction shows as light blue whereas a vertical structure appears as red. The brightness corresponds to the anisotropy, a higher anisotropy of the signal resulting in a brighter colour. As a consequence of this, completely isotropic scattering shows as black on the images. Additionally, the XVR and *μ*CT calculation results of the remaining samples P1–P8, excluding P4, are visualized in fig. 4 and fig. 5, respectively.

We calculated the average anisotropy over a square area in the centre of the bone as indicated in fig. 3(a). The values for XVR measurements and calculations based on *μ*CT data are listed in table 1. The correlation between the averaged anisotropy of both methods is illustrated in fig. 6(a). Each dot represents a pair of corresponding XVR/*μ*CT data from one of the eight bone cubes and one of the three available sample orientations. The linear regression line has coefficients and respective standard errors *a* = (0.018 ± 0.019) and *b* = (0.827 ± 0.089).

The mean orientation 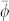 was calculated over the same area and the discrepancy 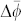 of every pair of measurement and calculation was averaged and a mean error of the orientation 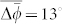 was found.

Furthermore, to demonstrate the preservation of the scattering information at very coarse resolutions, we rebinned the raw data before doing the stepping curve analysis and XVR processing. This corresponds to using a detector with pixel size of 508 *μ*m^2^ in the XVR measurement. The resulting images for one sample orientation are shown exemplarily in figures 3(d) and 3(h). For the *μ*CT calculation the the rebinning parameter was changed accordingly (also see Methods section). An average anisotropy was calculated for the rebinned XVR data and corresponding *μ*CT calculation and the correlation is shown in figure 6(b). The coefficients of the linear regression line are *a* = (0.022 ± 0.017) and *b* = (0.816 ± 0.085).

## Discussion

This study provides evidence that in trabecular bone the edges of the trabeculae in beam direction are indeed responsible for the formation of the X-ray vector radiography signal. We have shown a correct prediction of the preferred scattering orientation and anisotropy using *μ*CT data. Comparing the XVR data to those calculated from the *μ*CT measurement, the structure orientations and anisotropy values are in good agreement. It is to note that an area in which the trabeculae display an outstanding orientation compared to the bulk can both be seen in the XVR measurement and calculated by the presented method. Fig. 4(f) and fig. 5(f) depict XVR measurement and *μ*CT calculation results of a sample that possesses these scattering characteristics in direction 2. Comparison with the raw *μ*CT data reveals that there is indeed a strongly irregular layer of bone present throughout most of the sample in this particular area (see fig. 2(c)). The observed orientation can be traced back to the epiphyseal plate.

The quantitative analysis of the average anisotropy values shown in fig. 6 shows a good correlation between the two methods. We have shown that the correlation does not suffer as the pixelsize of the XVR data is increased and the presented model can explain the formation of the XVR signal even for very low resolutions. Furthermore, the anisotropy was calculated correctly not only for the average values but also for the distribution over an image. The distribution is not necessarily equal over the entire sample, e.g. the XVR measurement shown in fig. 3(e) exhibits a strong decline in anisotropy towards the right, a feature that is also found in the corresponding *μ*CT calculation (see fig. 3(b)). This study of anisotropy also confirms that our initial assumption of completely anisotropic scattering is valid, as isotropic scattering in the sample, i.e. from microspheres of size below the resolution of the *μ*CT scan would lower the anisotropy measured by the XVR setup substantially.

Limitations of the presented methods concern the fact that the dark field signal depends on both the linear absorption coefficient *μ* and the real part of the refractive index *n*^20^. In the calculations we assumed a parallel beam geometry, whereas a laboratory setup like the used XVR setup in general has a cone beam geometry (although with a small cone angle of 2°). In a more divergent geometry, a summation of the individual slices along one axis would not be correct any more and needs to be kept in mind for large samples. The presented study uses sufficiently small samples to warrant the assumption of a parallel beam addition, as the beam offset within the sample stays below the length scale of an individual pixel.

In conclusion, we have shown that the XVR signal of trabecular bone is primarily given by the existing trabecular structure in thick samples. This validation supports the idea of using X-ray vector radiography to gather supplementary information about the trabecular structures of femoral bone. Contrary to *μ*CT, this additional structural information can be obtained without the need of having to directly resolve the structures. The most striking advantage of XVR is that it is possible to acquire this scattering information at very coarse resolutions, comparable to DXA. Information about the bone microstructure, next to the bone density information obtained with DXA, is currently not easily accessible and, as we have shown, the scattering orientation and anisotropy of the XVR signal are two properties related to this microstructure. This shows up the possibilities of XVR radiography as a possible supplementary tool in the field of bone quality assessment, and we believe that XVR might help improve diagnosis of osteoporosis in the future.

## Methods

### X-ray absorption micro CT

**Figure 1:**
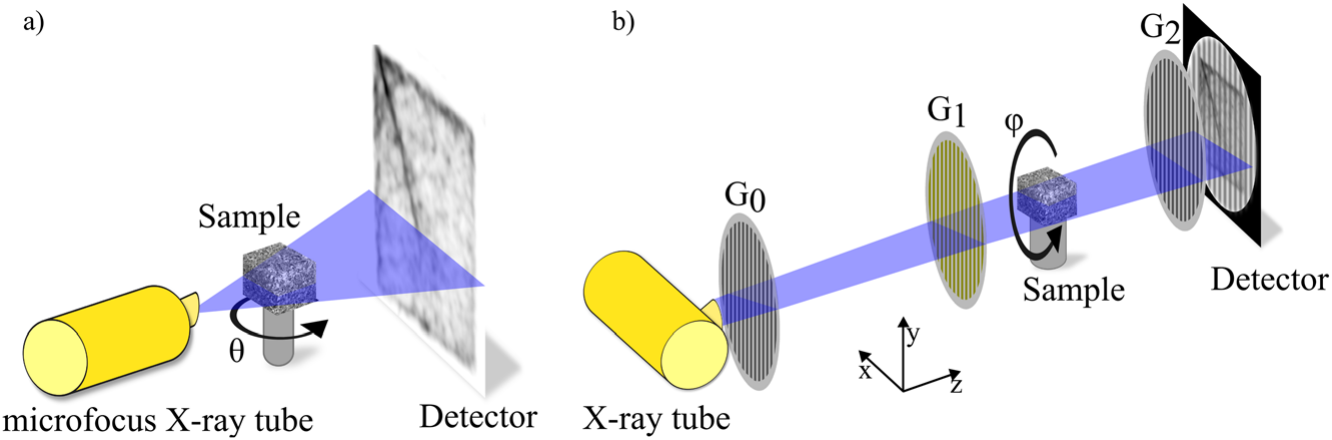
Schematics of the micro-CT system (a) and experimental XVR setup (b) used for the measurements. The micro-CT system utilizes a microfocus X-ray tube and divergent illumination to magnify a sample. The XVR setup employs a grating interferometer consisting of three gratings and is run with a conventional X-ray tube. Contrary to the micro-CT system, the sample is rotated in the plane perpendicular to the optical axis.

High resolution X-ray computed tomography (*μ*CT) data of the samples was obtained using a v|tome|x s 240 system by *GE Sensing & Inspection Technologies GmbH* (Germany), installed at the Technische Universität München. A sketch of the system is shown in figure 1(a).

**Figure 2:**
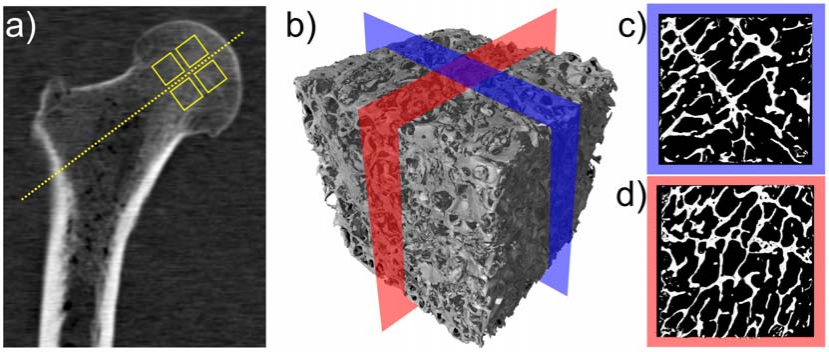
(a) Slice of a clinical CT of the upper part of an entire femur bone. The samples used in this study were prepared from the region around the rotational axis of the femoral head. This location is indicated by the four boxes. (b) Three-dimensional surface rendering of a high resolution *μ*CT of a 1 cm^3^ cube of human femur bone sample P7. (c,d) Two-dimensional cuts (10 mm × 10 mm) through both horizontal axes, taken from the centre region as indicated in (b).

For this purpose, the X-ray tube was operated at an acceleration voltage of 90 kV and current of 150 *μ*A. The geometry of the setup and resulting constraints in resolution lead to an isotropic voxel size of 18.53 *μ*m for every sample. This is sufficient to resolve the trabecular structure of human vertebral bones^21^. The duration of a single CT scan was approximately 70 minutes with 2000 projections being taken over 360°. Figs. 2(b–d) show a rendered 3D volume of sample P7 as well as two cuts through its middle. As can be seen, the trabecular structure is well resolved.

### X-ray vector radiography

The used setup is sketched in figure 1(b). A detailed description of a typical XVR setup and the measurement procedures can be found in e.g.^17^.

Our experiments were performed at the Technische Universität München at a laboratory setup operating a high power X-ray tube by *Comet AG*, Switzerland. For the experiments, the X-ray tube was operated at 60 kV and 30 mA with a 3.0 mm aluminum filter. The two absorption gratings consisted of 160 – 170 *μ*m thick gold lines on 500 *μ*m (G_0_) and 150 *μ*m (G_2_) thick silicon substrates, filled with SU-8 photoresist. Complementing the set of gratings is a phase grating (G_1_) made of 8 *μ*m nickel lines on a 200 *μ*m thick silicon substrate. This results in a phase shift of *π*/2 at the design energy of 45.7 keV. The absorption gratings had a periodicity of 10 *μ*m and the phase grating had a periodicity of 5.0 *μ*m. All gratings had a duty cycle of 0.5. The setup is built to operate at the first fractional Talbot distance with equidistant grating locations G_0_ to G_1_ and G_1_ to G_2_ of 92.7 cm. The samples were placed 28 cm behind G_1_ towards G_2_. A Varian PaxScan 2520D with a CsI scintillator and pixel pitch of 127 *μ*m was used as X-ray detector.

### Micro CT data processing

**Figure 3:**
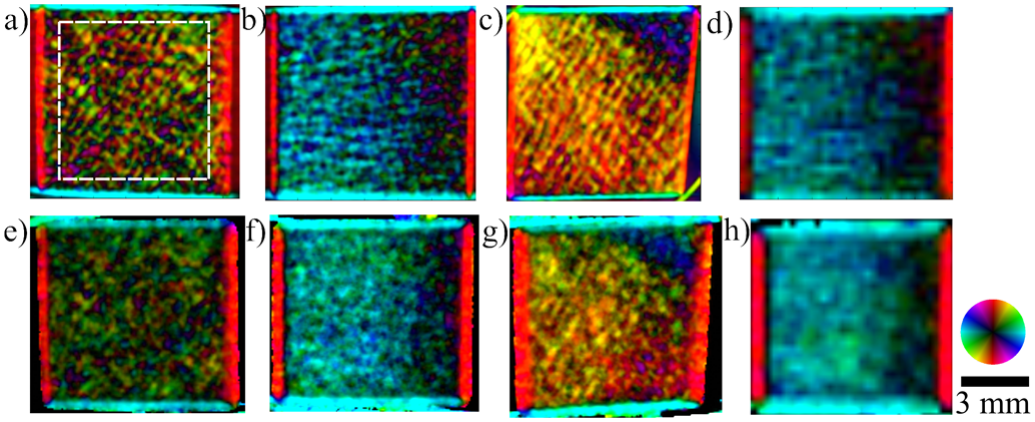
Calculation using *μ*CT data (a–d) and the corresponding X-ray Vector Radiography (XVR) images (e–h). Three orthogonal sample orientations of the same specimen are shown. The colour encodes the orientation of the strongest signal, the brightness codes the anisotropy. A structure only scattering into the horizontal plane appears red with maximum brightness, whereas a completely isotropic signal appears dark. A decline in anisotropy towards the right side can be seen in images (b) and (f) as a decline in brightness. Images (c) and (g) show closely matching orientations of *μ*CT calculation and XVR imaging for varying structural orientation. The area over which the anisotropy was averaged for a quantitative analysis is indicated in (a). The artefacts around the sample are caused by the sample holder. The effects of a greatly increased pixel size is shown for XVR measurements (h) and the corresponding *μ*CT calculation (d).

**Figure 4:**
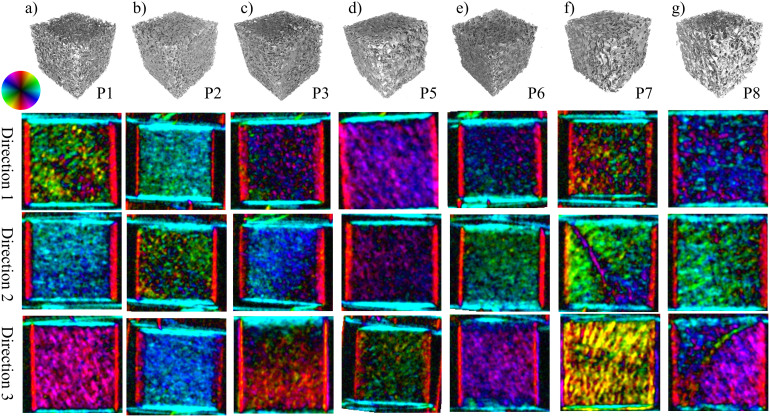
*μ*CT surface rendering and three XVR images from three orthogonal directions of specimen P1–P8 (a–g). Sample P4 is shown separately in figure 3. Various features can be seen in the different samples and orientations. For example, an area with structural orientation deviating by almost 90° from the surrounding area is visible in the samples shown in (f) and (g) from direction 2 and 3, respectively. The raw *μ*CT data show a strongly irregular trabecular structure present in these particular areas (also see fig. 2(b–d) for sample P7), which we believe corresponds to the epiphyseal plate.

**Figure 5:**
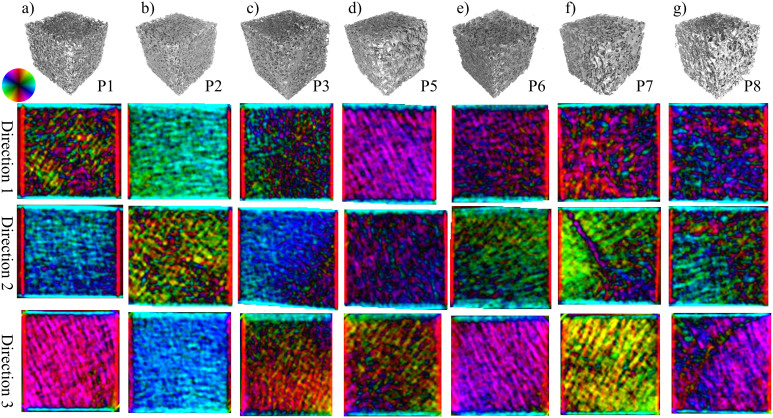
Shown is a *μ*CT rendering and the results of three calculations of the XVR signal, based on *μ*CT data. The samples and directions correspond to those shown in figure 4. The direction and anisotropy of the main structural orientation is encoded in the same way as for the XVR results in fig. 3.

**Figure 6:**
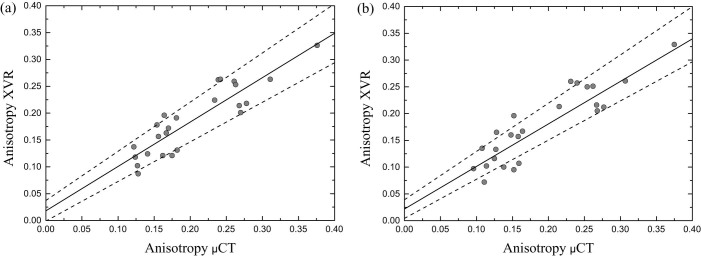
Mean anisotropy of XVR measurements plotted against mean anisotropy based on *μ*CT calculations. The area over which was averaged is indicated in figure 3(a). (a) The average anisotropy was calculated using the original XVR data with pixel size of 127 *μ*m^2^ and corresponding *μ*CT calculation. A linear fit line with coefficients *a* = (0.018 ± 0.019) and *b* = (0.826 ± 0.089) is added. (b) Rebinned XVR data corresponding to a very coarse detector pixel size of 508 *μ*m^2^ was used to calculate the mean anisotropy. The parameters used in the *μ*CT calculation was adjusted accordingly. The coefficients of the linear regression line are very similar to the original data presented in (a) with *a* = (0.022 ± 0.017) and *b* = (0.816 ± 0.085).

There is a variety of edge detection filters in image processing, we used the Roberts Cross Operator to locate differences in density which shall be introduced here briefly^22^: Let *μ_n_* be the slice with index n of the X-ray absorption data perpendicular to the beam direction in the XVR setup (z-axis). Then we can define for every slice the two gradients *G_x_* and *G_y_* as a convolution of the absorption data with the respective filter kernel: 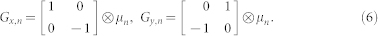
From these two gradients of *μ_n_* we can calculate the direction of biggest change *φ_n_*(*x*, *y*) and its magnitude *A_n_*(*x*, *y*): 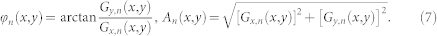
We assume that every edge within the sample produces a fully anisotropic signal (*a*_0_ = *a*_1_) in direction *φ_n_*(*x*, *y*) with magnitude *A_n_*(*x*, *y*). The XVR signal of the whole sample can be seen as a superposition of each slice's contribution^18^,^19^. Consequently, it is necessary to find a way to combine the calculated signals derived from the actual microstructure and originating from different slices in the propagation direction of the beam. It is also known that any XVR signal can be described as a linear superposition of various fully anisotropic signals *a*_1,*i*_ cos (2*φ*_,*i*_), which do not necessarily have the same phase, *φ*_,*i*_, or strength, *a*_,*i*_^18^,^19^: 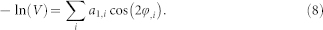
The factor 2 in the cosine's argument in eq.(8) accounts for the fact that due to the grating structure the signal does not vary with respect to a 180° rotation of the sample around the beam. With the previously mentioned assumption that scattering occurs fully anisotropic at each gradient and using the complex notation for a wave we can calculate a function that is expected to be proportional to the oscillating part of the XVR signal: 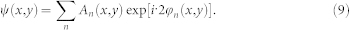
From these oscillating functions *ψ*(*x*, *y*) we can extract the main structural orientation one would derive from the XVR signal *φ_tot_*(*x*, *y*), which is equal to their argument in any given pixel *P*(*x*, *y*). The second important information one can obtain using XVR is the anisotropy of the signal. As defined earlier, it is the ratio of the oscillating part (*a*_1_) to the average signal (*a*_0_) after taking the negative logarithm of the recorded XVR signal. The absolute value of *ψ*(*x*, *y*) is proportional to *a*_1_(*x*, *y*), whereas the average part *a*_0_(*x*, *y*) is proportional to the sum of the amplitudes *A_n_*(*x*, *y*) along the beam axis under the assumption of completely anisotropic scattering. Therefore, we can state that: 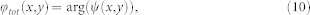

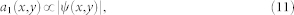

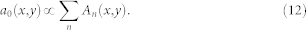
Since the proportionality constants in eq.(11) and eq.(12) depend only on the relative values given by the *μ*CT software and thus are the same, we can get the anisotropy by combining these equations: 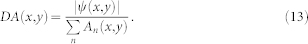
When comparing the anisotropy values calculated using this method with the ones directly obtained by a XVR measurement one needs to consider two effects which reduce the recorded anisotropy. Firstly, most pixelated detection mechanisms such as the used detector possess a certain lateral smearing of their response to a point signal, which can be described in terms of a point spread function (PSF). For this reason, a PSF was applied to the two-dimensional array of *ψ*(*x*, *y*) before calculating the orientation and anisotropy values by using the convolution operator (⊗): 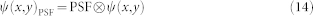
The unknown PSF of the detector was approximated with a two-dimensional Gaussian function of defined full width at half maximum (FWHM) at 10 pixels, corresponding to 185 *μ*m. Secondly, there is a difference in resolution between the *μ*CT and the XVR scan. For the purposes of this work, the resolution of the *μ*CT scans was much higher compared to the XVR setup. This needs to be considered in the calculations, as a single detector pixel of the XVR setup integrates the recorded intensity over an area of 127 × 127 *μ*m^2^. A binning of 5 × 5 pixels (20 × 20 pixels for the data set presented in Figure 6(b)) into a single pixel was applied on the complex valued function *ψ*(*x*, *y*) before calculating the orientation and anisotropy values. The size of the binning was chosen to account for the magnification introduced in the used XVR setup. Both of these effects can be seen as a lateral superposition of the signals in the individual pixels. These corrections affect mainly the calculated average anisotropy, the average orientation of the calculated signal remains nearly constant with varying parameters of the corrections. Samples with lower average anisotropy generally show a bigger decrease in anisotropy than those with a higher average anisotropy. This can easily be explained by the fact that samples with an overall lower anisotropy also had a greater variance in their scattering orientation. The reduction in anisotropy is stronger when signals with greatly different scattering orientations are summed up. As the width of the PSF is increased, a summation is done over a greater number of individual scattering orientations. Consequently, samples with a rapidly varying scattering orientation show a stronger decline in average anisotropy than very homogeneously scattering samples. In figure 3, orientation (a) of the sample resembles a very inhomogeneous scattering orientation, whereas orientation (b) shows a very homogeneous distribution of the scattering orientation. The overall average decrease of the average anisotropy for a PSF with FWHM of 5 pixels compared to the uncorrected case was 0.025 with a standard deviation of 0.013. For a PSF with FWHM of 10 pixels, these values nearly double with a decrease of 0.041 and standard deviation of 0.024, respectively.

The mean orientation of a sample was calculated by applying equations (9) and (10): 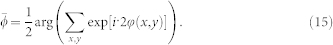
Using this notation, every pixel is weighted equally, although different weighting factors may be used, e.g. the average scattering strength *a*_0_.

## Author Contributions

F.S., A.M., G.P. and F.P. conceived the experiment. T.B., E.G.C. and J.S.B. supplied and prepared the samples. P.B.N. provided clinical CT data to aid with sample preparation. E.E. performed the measurements. F.S. performed the data analysis with significant input from A.M., G.P. and F.P. The paper was written by F.S., A.M. and F.P. with input from all co-authors.

## Figures and Tables

**Table 1:** Mean anisotropy values of XVR measurements and *μ*CT calculations, the values were derived from an area as indicated in figure 3(a) for each sample. The three orthogonal directions that were measured are indicated as 1,2 and 3, consistent with the notation used in figures 4 and 5

Sample		P1	P2	P3	P4	P5	P6	P7	P8
calculation	1	0.156	0.261	0.124	0.162	0.268	0.141	0.182	0.170
	2	0.239	0.175	0.242	0.164	0.127	0.122	0.181	0.154
	3	0.311	0.376	0.167	0.278	0.128	0.270	0.263	0.234
XVR	1	0.156	0.259	0.118	0.121	0.214	0.124	0.131	0.172
	2	0.262	0.121	0.263	0.196	0.102	0.137	0.191	0.178
	3	0.263	0.326	0.163	0.218	0.087	0.201	0.253	0.224
